# The metabolism and de-bromination of bromotyrosine *in vivo*

**DOI:** 10.1016/j.freeradbiomed.2015.11.030

**Published:** 2016-01

**Authors:** Ali R. Mani, José C. Moreno, Theo J. Visser, Kevin P. Moore

**Affiliations:** a Division of Medicine, Royal Free Campus, University College London (UCL), Rowland Hill Street, NW3 2PF London, UK; b Thyroid Molecular Laboratory, Institute for Medical and Molecular Genetics (INGEMM), La Paz University Hospital, Madrid, Spain; c Department of Internal Medicine, Erasmus University Medical Centre, Rotterdam, The Netherlands

**Keywords:** Bromotyrosine, 3-Bromo-4-hydroxyphenylacetic acid, De-halogenation, 4-Hydroxyphenylacetic acid, Urine

## Abstract

During inflammation, leukocyte-derived eosinophil peroxidase catalyses the formation of hypobromous acid, which can brominate tyrosine residues in proteins to form bromotyrosine. Since eosinophils are involved in the pathogenesis of allergic reactions, such as asthma, urinary bromotyrosine level has been used for the assessment of children with asthma. However, little is known about the metabolism and disposition of bromotyrosine *in vivo*. The aim of this study was to identify the major urinary metabolites formed during bromotyrosine metabolism and to develop mass spectrometric methods for their quantitation. Deuterium-labeled bromotyrosine was synthesized by deuterium exchange. [D_3_]bromotyrosine (500 nmole) was injected intraperitoneally into Sprague-Dawley rats and urine was collected for 24 h in a metabolic cage. ^13^C-labeled derivatives of bromotyrosine and its major urinary metabolite were synthesized and used as internal standards for quantitation. Following solid phase extraction, urine samples were derivatized to the pentafluorobenzyl ester, and analyzed using isotope dilution gas chromatography and negative-ion chemical ionization mass spectrometry. A novel brominated metabolite, 3-bromo-4-hydroxyphenylacetic acid (bromo-HPA), was identified as the major brominated metabolite of bromotyrosine. Bromo-HPA only accounted for 0.43±0.04% of infused [D_3_]bromotyrosine and 0.12±0.02% of infused [D_3_]bromotyrosine was excreted in the urine unchanged. However, ~1.3% (6.66±1.33 nmole) of infused [D_3_]bromotyrosine was excreted in the urine as the de-brominated metabolite, [D_3_]4-hydroxyphenylacetic acid, which is also a urinary metabolite of tyrosine in mammals. We also tested whether or not iodotyrosine dehalogenase can catalyse de-bromination of bromotyrosine and showed that iodotyrosine dehalogenase is able to de-brominate free bromotyrosine *in vitro*. We identified bromo-HPA as the main brominated urinary metabolite of bromotyrosine in rats. However, de-halogenation of bromotyrosine is the major metabolic pathway to eliminate free brominated tyrosine *in vivo*.

## Highlights

• De-bromination of bromotyrosine occurs during metabolism of free bromotyrosine.
• 4-Hydroxyphenylacetic acid is the major urinary metabolite of bromotyrosine in rats.
• 3-Bromo-4-hydroxyphenylacetic acid was identified as a brominated metabolite of bromotyrosine.
• Iodotyrosine dehalogenase is able to de-brominate free bromotyrosine *in vitro*.

## Graphical abstract

**Figure:**
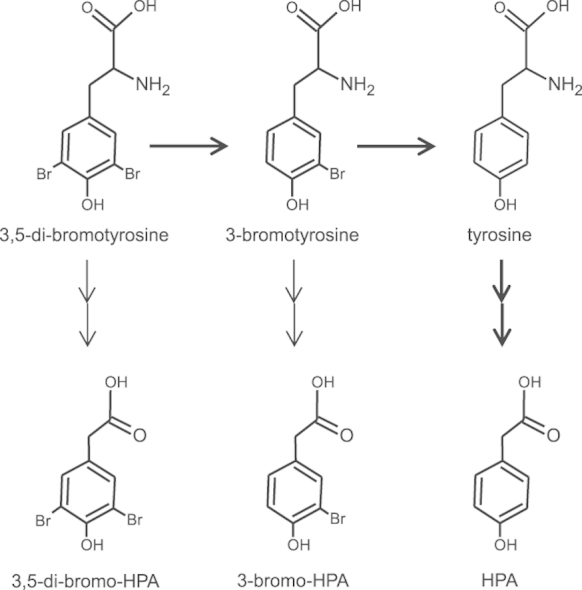

## 1 Introduction

Eosinophils play an important role in the mammalian immune system during allergic reactions, as well as in immune responses against extracellular parasites. Following activation, eosinophils release effectors, such as cationic proteins and eosinophil peroxidase, that lead to host defense and/or tissue damage [1]. Eosinophil peroxidase is a halide peroxidase that preferentially uses bromide to generate hypobromous acid, even at physiological halide concentrations where chloride is almost 1000-fold greater than bromide concentrations [1]. Hypobromous acid is a brominating agent that can brominate nucleophilic aromatic compounds, such as tyrosine [2], [3]. Wu et al., reported that 3-bromotyrosine and 3,5-di-bromotyrosine are detectable following eosinophil peroxidase-induced protein oxidation [2]. Likewise, neutrophil-derived myeloperoxidase uses chloride, bromide and nitrite ions to generate halogenating and nitrating agents that lead to the formation of 3-chlorotyrosine, 3-bromotyrosine and 3-nitrotyrosine [4], [5], [6]. Therefore, halogenated tyrosine residues are commonly used to quantify leukocyte-mediated damage in diseased tissues [7], [8]. However, one of the major disadvantages of measuring bromotyrosine or chlorotyrosine is that oxidized proteins undergo proteolysis, and the resulting free halogenated amino acids are metabolized and excreted in the urine [9].

Since eosinophils play an important role in the pathogenesis of allergic asthma, quantitation of urinary bromotyrosine levels has been used as a non-invasive biomarker for the monitoring of children with asthma [10], [11], [12]. Thus, Wedes et al. demonstrated that urinary bromotyrosine levels predict the risk of future asthma exacerbations in children [11]. Moreover, Cowan et al., have recently reported that urinary bromotyrosine levels can be used to predict the responsiveness to inhaled corticosteroid therapy in asthmatic patients [12]. Although urinary bromotyrosine measurements have been used for the assessment of eosinophil activation, very little is known about the metabolism and disposition of bromotyrosine *in vivo*. This is surprising, given the fact that major urinary metabolites of chlorotyrosine and nitrotyrosine have been identified [9], [13], [14] and their measurement has been employed for assessment of systemic inflammation *in vivo* [15], [16]. Thus, it is known that chlorotyrosine is metabolized to 3-chloro-4-hydroxyphenylacetic acid (chloro-HPA) and 4-hydroxyphenylacetic acid (HPA), the major urinary metabolites of chlorotyrosine *in vivo* [9]. We previously found that, following infusion of deuterium-labeled chlorotyrosine, the urinary excretion rate of deuterium-labeled chloro-HPA and HPA was 3.3- and 35.6-fold greater than the un-metabolized chlorotyrosine, respectively [9]. Likewise, quantitation of the urinary metabolites of bromotyrosine may provide a better, non-invasive assessment of bromination reactions *in vivo* than the urinary concentration of un-metabolized bromotyrosine. In the present study, we used mass spectrometry in order to identify the major urinary metabolites of bromotyrosine and di-bromotyrosine.

## 2 Materials and methods

### 2.1 Chemicals

All chemicals were purchased from Sigma-Aldrich, unless stated otherwise. [^13^C_9_]Tyrosine, [D_4_]acetic acid, D_2_O, and deuterium chloride (37% in D_2_O) were purchased from Cambridge Isotope Laboratories (Andover, MA).

### 2.2 Animals

Male Sprague-Dawley rats (270–300 g) were obtained from the Comparative Biology Unit at University College London. Animal procedures were in accordance with the Home Office, UK guidelines.

### 2.3 Synthesis of deuterium-labeled compounds

[D_3_]Bromotyrosine was synthesized by deuterium exchange as described [9], [14], using authentic bromotyrosine as starting material. In brief, 50 mg of bromotyrosine were dissolved in a mixture of [D_4_]acetic acid and D_2_O, and the solvent was evaporated in a stream of nitrogen at 90 °C. This procedure was repeated twice to remove active protons. The resulting residue was dissolved in a mixture of [D_4_]acetic acid, D_2_O and deuterium chloride. The solution was sealed in an acid-digestion bomb and heated in an autoclave at 190 °C for 8 h [9], [14]. The resulting product was dissolved in 0.1% (v/v) trifluoroacetic acid (TFA)/water (adjusted to pH 5.0 with ammonia solution) and extracted on a LC18 reverse-phase column, pre-washed with 2 ml of methanol and 5 ml of 0.1% (v/v) TFA/water (pH 5.0). The products were washed with water, and the deuterated products eluted with 4 ml of 30 % (v/v) methanol in water. The products were purified further by thin-layer chromatography [14], and their concentrations determined against known amounts of unlabeled standards by GC/MS (see below).

### 2.4 Synthesis of ^13^C-labeled internal standard for the measurement of bromotyrosine, 4-hydroxyphenylacetic acid (HPA) and 3-bromo-4-hydroxyphenylacetic acid (bromo-HPA)

[^13^C_9_]Bromotyrosine was synthesized by the reaction of [^13^C_9_]tyrosine with hypobromous acid (HOBr) at room temperature for 1 h. HOBr was prepared freshly by adding HOCl to NaBr as described [17]. The products were purified using LC18 solid phase extraction, followed by HPLC. [^13^C_8_]4-hydroxyphenylacetic acid (HPA) was synthesized following deamination and decarboxylation of [^13^C_9_]tyrosine, using Taiwan cobra venom as described previously [14]. [^13^C_8_]3-Bromo-4-hydroxyphenylacetic acid (bromo-HPA) was synthesized and purified by bromination of [^13^C_8_]HPA in the same way as bromotyrosine, and the concentrations of ^13^C-labeled standards determined against known amounts of unlabeled standards by GC/MS.

### 2.5 Measurement of bromotyrosine and tyrosine by GC/MS

^13^C-Labeled internal standard (5 ng) was added to 20 µl of rat urine and diluted to 1 ml of total volume with 0.1 % (v/v) TFA/water (pH 5.0). The samples were extracted using LC18 reverse phase column, as described above. Bromotyrosine and tyrosine were derivatized with ethyl heptafluorobutyrate and silylated with tert-butyldimethylsilyl, as described [9], [17], [18]. Derivatized samples were dried under nitrogen, and redissolved in 20 µl of n-undecane. Samples were applied to a GC equipped with a 15-m DB-1701 (J&W Scientific, Folsom, CA) capillary column (0.25-mm internal diameter, 0.25-mm film thickness), interfaced with a mass spectrometer (Trio 1000; Fisons Instruments, Beverly, MA). The ion source and interface temperatures were set at 200 and 320 °C, respectively. Samples were analyzed in negative-ion chemical ionization mode with ammonia as the reagent gas, using 1 µl of each sample for injection. The initial column temperature was maintained at 150 °C for 1 min increasing to 300 °C at 20 °C/min. Ions were monitored at 489, 492, and 498 mass units for authentic, [D_3_] and [^13^C_9_]bromotyrosine, respectively (Table 1). For measurement of tyrosine, samples were monitored at 407, 410 and 415 m/z for authentic, [D_3_]tyrosine and [^13^C_9_]tyrosine, respectively. Concentrations were calculated by ratio to known ^13^C_9_-labeled internal standards.

### 2.6 Measurement of bromo-HPA and HPA by GC/MS

^13^C-Labeled internal standard (5 ng) was added to 20 µl of rat urine and diluted to 1 ml with 0.1 % (v/v) TFA/water (pH 5.0). The samples were extracted using LC18 reverse-phase column as described above. The samples were derivatized to the pentafluorobenzyl ester by the addition of 20 µl of di-isopropyl ethylamine in acetonitrile and 40 ml of 10 % (v/v) pentafluorobenzyl bromide in acetonitrile for 1 h at room temperature (21–23 °C), dried under nitrogen, and redissolved in 20 ml of n-undecane for GC/MS analysis [9], [14]. The GC/MS set up was as described above, and ions were monitored at 409, 412, and 417 mass units for authentic, [D_3_] and [^13^C_8_]bromo-HPA, respectively, with single-ion monitoring (Table 1). For measuring HPA, samples were monitored at 331, 334, and 339 mass units for authentic, [D_3_] and [^13^C_8_]HPA, respectively. The concentrations were calculated from the known ^13^C_8_-labeled internal standards.

### 2.7 Metabolism of di-bromotyrosine *in vivo*

A similar method used by Ohshima et al. [13] was used for identification of urinary metabolites of di-bromotyrosine. Male Sprague-Dawley rats (*n*=3 in each group) were given intraperitoneal injection of either di-bromotyrosine or tyrosine (30 µmol). Urine was collected in metabolic cages for 24 h. Urine samples were acidified to pH 1.0 with HCl and extracted with an equivalent volume of ethyl acetate. The concentrated extracts were derivatized to the pentafluorobenzyl ester by the addition of 20 µl of di-isopropyl ethylamine in acetonitrile and 40 ml of 10% (v/v) pentafluorobenzyl bromide in acetonitrile for 1 h at room temperature (21–23 °C), dried under nitrogen, and re-dissolved in 20 ml of n-undecane for full scan GC/MS analysis [9].

### 2.8 Metabolism of [D_3_]bromotyrosine *in vivo*

Male Sprague-Dawley rats (*n*=3 in each group) were given a single intraperitoneal injection of [D_3_]bromotyrosine (0.50 µmol) [9]. The animals were then transferred to standard metabolic cages, and urine samples were collected for 24 h. The levels of deuterated bromotyrosine, tyrosine, bromo-HPA, and HPA were measured in the collected urine samples, as described above.

### 2.9 De-bromination of [D_3_]bromotyrosine by cultured hepatoblastoma (HepG2) cells

HepG2 cells (ECACC, Wiltshire, UK) were maintained in MEM (minimum Eagle's medium) supplemented with 10% fetal calf serum. Monolayer HepG2 cells were plated in 175 cm^3^ vented tissue culture flasks until 70% confluent, when they were incubated with [D_3_]bromotyrosine (final concentration 25 µM). 24 h after incubation with [D_3_]bromotyrosine, cells were washed two times with PBS and were detached from the flask using a cell scraper. Detached cells were washed and re-suspended in 2 ml of PBS. Cell suspensions were subjected to protein precipitation by adding 1 ml of ice-cold chloroform/methanol (2:1, v/v) solution. After centrifugation (1000×*g* for 20 min), protein precipitates were washed with methanol and lyophilized under vacuum for assessment of protein-bound [D_3_]bromotyrosine and [D_3_]tyrosine as described [9], [17], [18]. In brief, 1–2 mg freeze-dried proteins were hydrolyzed in alkaline condition (1 ml of 4 M NaOH for 16 h at 120 °C) in presence of 20 ng and 1 µg of [^13^C_9_]bromotyrosine and [^13^C_9_]tyrosine, respectively, as internal standard. The samples were extracted using LC18 and ENV+ (Jones Chromatography, UK) reverse phase columns. The samples were then derivatized with ethyl heptafluorobutyrate and silylated with tert-butyldimethylsilyl, and monitored for ion masses of 492, 498, 410, 415 for [D_3_]bromotyrosine, [^13^C_9_]bromotyrosine, [D_3_]tyrosine and [^13^C_9_]tyrosine, respectively (Table 1) using negative-ion chemical ionization GC/MS [9], [17], [18]. The experiment was repeated three times. All data are expressed as mean±standard error.

### 2.10 Interaction of bromotyrosine and iodotyrosine dehalogenase (DEHAL) *in vitro*

Iodotyrosine dehalogenase 1 (DEHAL1) was obtained from HEK293 cells transfected with DEHAL1 cDNA, using a pcDNA3.1 expression vector [9], [19]. To determine whether bromotyrosine, di-bromotyrosine or bromo-HPA interact with iodotyrosine dehalogenase, deiodinase activity was measured based on radioiodide formation from [125I]iodotyrosine. Incubation mixtures contained [125I]iodotyrosine (∼100,000 cpm mixed with 0.1 μM unlabeled iodotyrosine), DEHAL1 enzyme, and varying amounts of iodotyrosine, bromotyrosine, di-bromotyrosine, bromp-HPA and tyrosine (0.01–100 μM final concentration) in 100 μl of PBS containing NADPH (100 μM), EDTA (2 mM), and dithiothreitol (10 mM) [9], [19]. Mixtures were incubated in duplicate for 60 min at 37 °C, and the reactions were stopped by addition of 0.9 ml of 10% acetic acid on ice. The radioiodide in the mixture was separated from the remaining [125I]iodotyrosine with ion exchange chromatography on Dowex columns. The columns were washed with 10 % acetic acid and eluted with 8 ml of 1 mM ammonia solution. Blank incubations without enzyme were used to correct for non-enzymatic deiodination. To determine whether bromotyrosine or di-bromotyrosine could act as a substrate of DEHAL1, the brominated or iodinated tyrosine derivatives (1 μM) were incubated with DEHAL1 in 100 μl of PBS containing NADPH (100 μM), EDTA (2 mM), and dithiothreitol (10 mM). Mixtures were then incubated in duplicate for 60 min at 37 °C, and the reaction was stopped by addition of 1 ml of 10 % acetic acid on ice. Substrate levels were measured using HPLC as described [9]. Cell lysates from HEK293 cells transfected with empty pcDNA3.1 plasmid were used as control. In experiments where di-bromotyrosine, or di-iodotyrosine was used as substrate, concentrations of mono-bromotyrosine and mono-iodotyrosine were measured as a product of de-halogenation.

## 3 Results

### 3.1 Structural confirmation of bromotyrosine and tyrosine by GC/MS

**Fig. 1:**
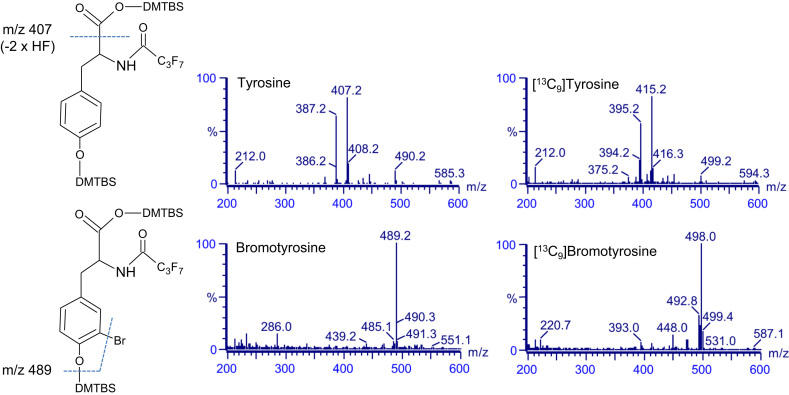
Structures and spectra from negative-ion chemical ionization scans of the ethyl heptafluoro and tert-butyldimethylsily derivatives of authentic and ^13^C-labeled tyrosine (upper panel) and 3-bomotyrosine (lower panel).

For GC/MS analysis of bromotyrosine and tyrosine, the amine group was derivatized with ethyl heptafluorobutyrate, and the hydroxyl and carboxylic groups were silylated with tert-butyldimethylsilyl and the products analyzed by negative-ion chemical ionization mass spectrometry. Full scan mass spectra obtained for these molecules are shown in Fig. 1. The major fragment ion of tyrosine corresponds to loss of COO-tert-butyl-dimethylsilyl, which results in a dominant ion at *m*/*z* 407 [17], [18]. The major fragment ion for bromotyrosine has an *m*/*z* of 489, which corresponds to loss of tert-butyldimethylsilyl and bromide [17]. Fig. 1 also shows the mass spectrum of derivatized ^13^C-labeled tyrosine and bromotyrosine. Full scan analysis of derivatized deuterated bromotyrosine showed that the synthesized compound had a dominant ion at *m*/*z* 492, which was 3 mass units heavier than the authentic bromotyrosine, and which corresponded to [D_3_]bromotyrosine.

### 3.2 Structural confirmation of di-bromo-HPA, bromo-HPA and HPA by GC/MS

**Fig. 2:**
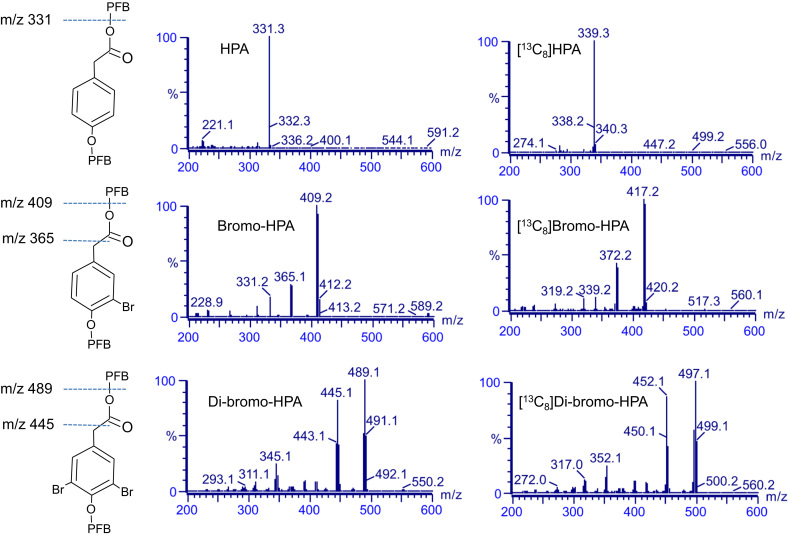
Structures and spectra from negative-ion chemical ionization scans of the pentafluorobenzyl derivative of authentic and ^13^C-labeled 4-hydroxyphenylacetic acid (HPA, upper panel), 3-bromo-4-hydroxyphenylacetic acid (bromo-HPA, middle panel) and 3,5-di-bromo-4-hydroxyphenylactic acid (di-bromo-HPA, lower panel).

The structures of the pentafluorobenzyl ester derivatives of HPA, bromo-HPA and di-bromo-HPA are shown in Fig. 2. Analysis of the pentafluorobenzyl ester derivatives of these compounds by full scan mode showed that HPA has a dominant ion at *m*/*z* 331 (M-181), which is due to the loss of a pentafluorobenzyl ester group. As shown in Fig. 2, bromo-HPA had a dominant ion at *m*/*z* 409 (M-181, loss of one pentafluorobenzyl ester group). Di-bromo-HPA had a dominant ion at *m*/*z* 489 (M-181), which is also caused by the loss of a pentafluorobenzyl ester group (Fig. 2). On the basis of these results, all further work was focused on developing a method to quantitate di-bromo-HPA, bromo-HPA and HPA using the pentafluorobenzyl ester derivative with selective ion monitoring at 489, 409 and 331 mass units for di-bromo-HPA, bromo-HPA and HPA, respectively. Fig. 2 also shows the mass spectra of derivatized ^13^C-labeled derivatives of HPA and brominated HPA. As shown in this figure, the synthesized compounds had dominant ions, which were 8 mass units heavier than the authentic compounds corresponding to [^13^C_8_]di-bromo-HPA, [^13^C_8_]bromo-HPA and [^13^C_8_]HPA.

### 3.3 Metabolism of di-bromotyrosine and bromotyrosine

**Fig. 3:**
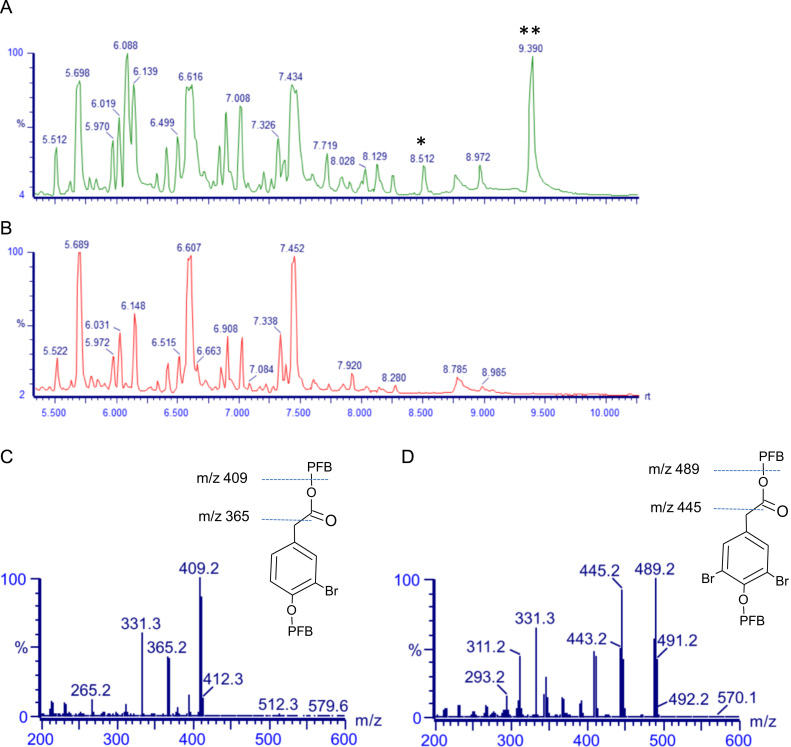
Gas chromatogram of urine samples analyzed with full scan mass spectrometry after derivatization with pentafluorobenzyl bromide. The analysis of urine samples from rats given 3,5-di-bromotyrosine (A) showed two consistent additional peaks on the chromatogram compared with urine samples of tyrosine treated rats (B). The mass spectra of these peaks are identical to those of the pentafluorobenzyl derivatives of 3-bromo-4-hydroxyphenylactic acid (C) and 3,5-di-bromo-4-hydroxyphenylactic acid (D).

**Fig. 4:**
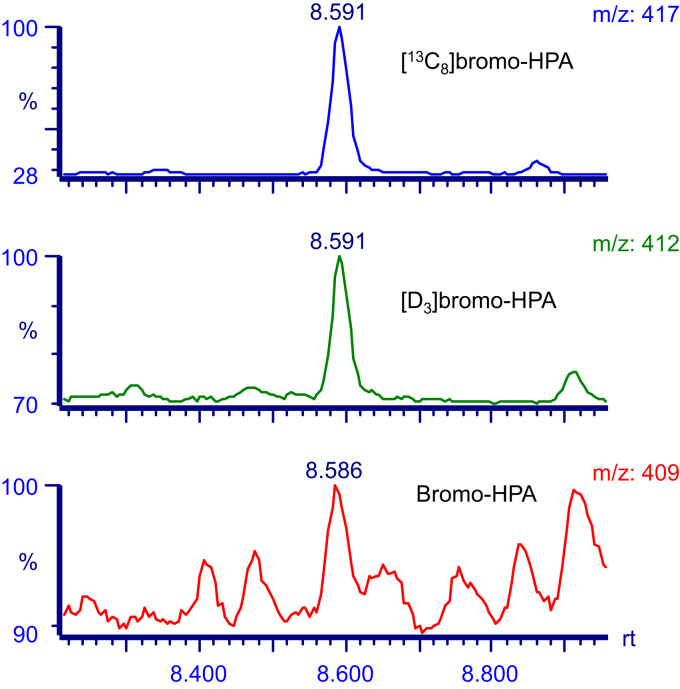
Chromatogram of a representative urine sample obtained from a rat injected with [D_3_]bromotyrosine. Formation of deuterated 3-bromo-4-hydroxyphenylacetic acid ([D_3_]bromo-HPA) represented as a distinguished peak that was detected with *m*/*z* 412 (middle panel). The values of *m*/*z* 409 (lower panel) and *m*/*z* 417 (upper panel) represent authentic bromo-HPA and ^13^C-labeled internal standard respectively.

Analysis of urine extracts from rats given di-bromotyrosine showed two main additional peaks on the GC, compared with urine obtained from rats given tyrosine alone (Fig. 3A and B). Full scan mass spectra of these two peaks are shown in Figs. 3C and 4D. These peaks were identified as di-bromo-HPA (the peak with retention time of 9.39 min) and bromo-HPA (the peak with retention time of 8.51 min). In separate experiments, we incubated the urine samples with alkaline phosphatase (from calf intestine, 300 U/ml) and sulfatase (300 U/ml, from *Helix pomatia*) to break sulfate and phosphate groups, in order to enhance derivatization of the metabolites. Although incubation of urine samples with phosphatase and sulfatase enhanced the appearance of bromo-HPA peaks, we were unable to identify any other new metabolites when the chromatograms of di-bromotyrosine and tyrosine treated rat urine samples were compared.

In further experiments, we injected rats with 0.5  µmole of [D_3_]bromotyrosine and used GC/MS to quantify urinary concentrations of [D_3_]bromotyrosine, [D_3_]tyrosine, [D_3_]bromo-HPA and [D_3_]HPA by isotope dilution mass spectrometry. As shown in Fig. 4, a peak with *m*/*z* of 412 was detectable in the urine of [D_3_]bromotyrosine treated rats, corresponding to the formation of [D_3_]bromo-HPA. This peak was not detectable in normal rat urine (data not shown). Urinary [D_3_]bromo-HPA was quantified using a ^13^C-labeled internal standard that we synthesized. [D_3_]bromo-HPA accounted for 0.43±0.04% of infused [D_3_]bromotyrosine. 0.12±0.02% of infused [D_3_]bromotyrosine was excreted in the urine unchanged. [D_3_]tyrosine was undetectable in rat urine samples. However ~1.3% (6.66±1.33 nmole) of infused [D_3_]bromotyrosine was excreted in the urine as the de-brominated metabolite, [D_3_]HPA, which is the main urinary metabolite of tyrosine in mammals, which suggests that dehalogenation of bromotyrosine occurs *in vivo* ( Table 2).

### 3.4 De-bromination of [D_3_]bromotyrosine by cultured HepG2 cells

**Fig. 5:**
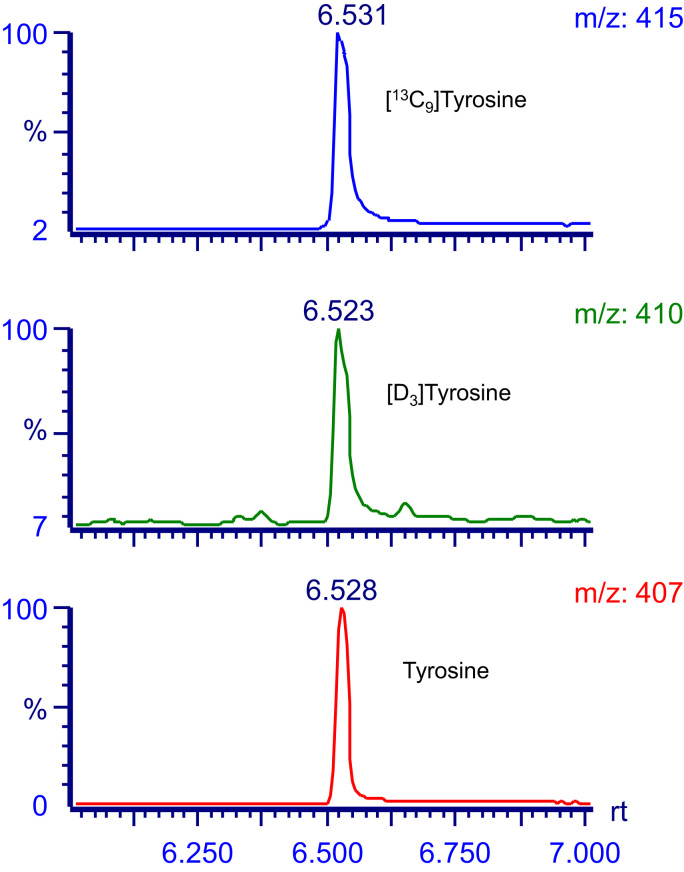
Chromatogram of the proteolyzed cell lysate after incubation of HepG2 cells with [D_3_]bromotyrosine (25 µM). Incorporation of [D_3_]tyrosine in the cellular proteins is represented as a peak that was detected with *m*/*z* 410. The values of *m*/*z* 407 and *m*/*z* 415 represent authentic tyrosine and ^13^C-labeled internal standard respectively.

We incubated deuterium-labeled bromotyrosine with cultured HepG2 cells and looked at incorporation of deuterated bromotyrosine ([D_3_]bromotyrosine) and tyrosine ([D_3_]tyrosine) into cellular proteins. As shown in Fig. 5, we could detect deuterated tyrosine (but not deuterated bromotyrosine) in the cell fraction obtained from HepG2 cells following incubation with [D_3_]bromotyrosine. This suggests that bromotyrosine is not incorporated directly into proteins, but can be de-brominated to tyrosine, which is incorporated. Thus, using ^13^C-labeled tyrosine as internal standard, we could show that 6.68±0.52 nmole of [D_3_]tyrosine is detectable in cellular proteins following incubation of the cells for 24 h with 250 nmoles of [D_3_]bromotyrosine (25 µM solution, total volume 10 ml).

### 3.5 Interaction of DEHAL1 and brominated tyrosine derivatives in vitro

**Fig. 6:**
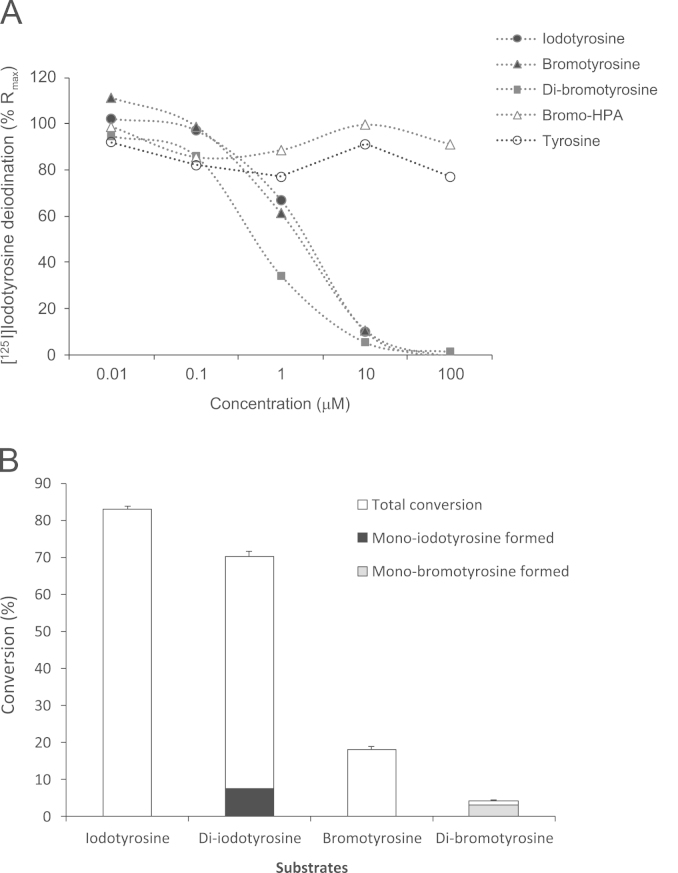
(A) The effect of di-bromotyrosine, bromotyrosine, bromo-HPA, iodotyrosine, and tyrosine on iodotyrosine dehalogenase 1 (DEHAL1)-induced deiodination of radiolabelled iodotyrosine. (B) The effect of DEHAL1 on dehalogenation of iodotyrosine, di-iodotyrosine, bromotyrosine and di-bromotyrosine in the presence of NADPH and dithiothreitol.

As shown in Fig. 6A, both bromotyrosine and di-bromotyrosine could inhibit DEHAL1-induced deiodinase activity in a concentration-dependent manner. Neither tyrosine nor bromo-HPA showed the ability to inhibit deiodination induced by DEHAL1. We then tested whether or not bromotyrosine might serve as a substrate for DEHAL1 dehalogenase. DEHAL1 converted 83% and 18% of iodotyrosine and bromotyrosine, respectively (Fig. 6B). Likewise, DEHAL1 converted 70% and 4% of di-iodotyrosine and di-bromotyrosine, respectively. As shown in Fig. 6B, incubation of di-iodotyrosine and di-bromotyrosine with DEHAL1 was associated with formation of mono-iodotyrosine (7%) and mono-bromotyrosine (3%). No dehalogenation occurred when the substrates were incubated with control cell lysates transfected with empty pcDNA3.1 plasmid.

## 4 Discussion

Eosinophil peroxidase is a haloperoxidase that preferentially uses bromide over chloride for generating hypobromous acid to kill extracellular parasites. Hypobromous acid can readily brominate biomolecules such as tyrosine and uracil to form 3-bromotyrosine and 5-bromouracil [2], [3].

**Fig. 7:**
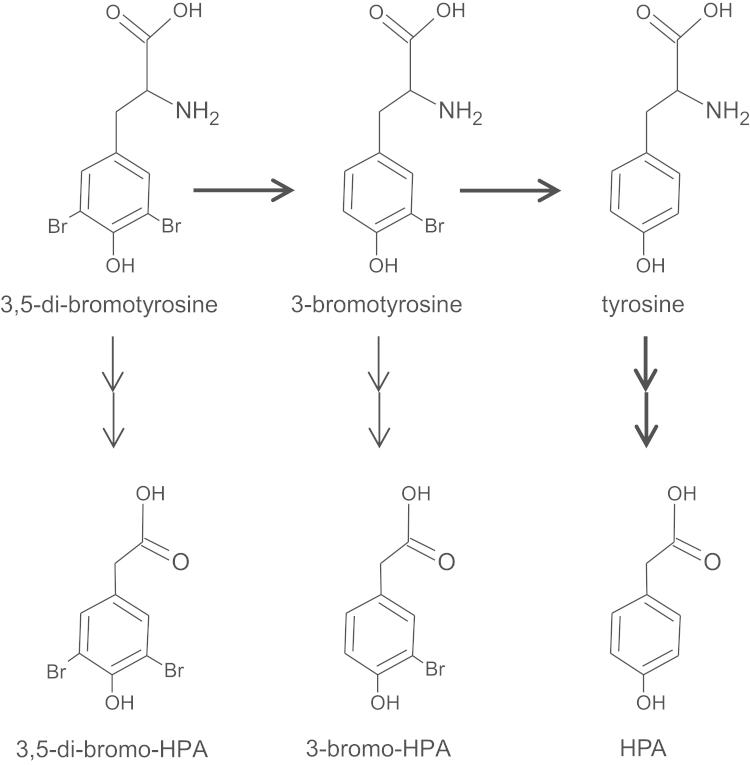
The metabolism of 3,5-di-bromotyrosine and 3-bromotyrosine *in vivo*. Both 3,5-di-bromotyrosine and 3-bromotyrosine can undergo deamination and decarboxylation to form 3,5-di-bromo-4-hydroxyphenlacetic acid (3,5-di-bromo-HPA) and 3-bromo-4-hydroxyphenlacetic acid (3-bromo-HPA). In addition, both 3,5-di-bromotyrosine and 3-bromotyrosine undergo de-bromination to form de-brominated metabolites such as 4-hydroxypenylacetic acid (HPA).

Since eosinophil activation is involved in the pathogenesis of eosinophilic asthma, recent studies have focused on measuring urinary concentrations of bromotyrosine as a potential biomarker for assessment of patients with asthma [10], [11]. Although this approach is interesting within the context of developing a non-invasive biomarker for screening and monitoring of patients with asthma, it is likely that bromotyrosine is metabolized *in vivo* and the urinary level of un-metabolized bromotyrosine may reflect only a small fraction of brominated tyrosine that is formed during eosinophil activation. It is well known that tyrosine and its derivatives such as nitrotyrosine and chlorotyrosine undergo deamination and decarboxylation to form HPA, 3-nitro-HPA and 3-chloro-HPA [9], [13], [14]. In addition, we and others have also shown that chlorotyrosine and iodotyrosine are readily de-halogenated *in vivo* [8], [19], [20]. In the present study, we observed two extra peaks following the administration of a single dose of di-bromotyrosine when compared with the urinary chromatograms of tyrosine injected animals. The mass spectra of these two peaks were compatible with di-bromo-HPA and mono-bromo-HPA (Fig. 3). Both of these metabolites are deaminated and decarboxylated metabolites (Fig. 7). Furthermore, formation of mono-bromo-HPA indicates that de-bromination reaction also occurs during metabolism of di-bromotyrosine *in vivo*. To make sure that the formation of mono-bromotyrosine is not an artifact, we measured the purity of injected di-bromotyrosine and excluded the possibility of contamination with mono-bromotyrosine (data not shown).

To further confirm these results we synthesized deuterium-labeled and ^13^C-labeled bromotyrosine and its urinary metabolites. Since large doses of bromotyrosine may saturate the metabolic enzymes, we injected a low dose (500 nmole) of [D_3_]bromotyrosine and measured urinary concentrations of [D_3_]bromo-HPA, [D_3_]HPA as well as un-metabolized [D_3_]bromotyrosine. Our results confirm the formation of both brominated and de-brominated metabolites ([D_3_]bromo-HPA and [D_3_]HPA). We also demonstrated that the urinary excretion rate of deuterium-labeled bromo-HPA and HPA were 3.6- and 10.7-fold greater than for un-metabolized bromotyrosine, respectively. Thus, our studies confirm that the urinary excretion of bromo-HPA is markedly higher than that of un-metabolized bromotyrosine, and bromo-HPA may therefore be a better marker for the non-invasive assessment of bromotyrosine formation and thus eosinophil activation, *in vivo*, compared with un-metabolized bromotyrosine. Recent reports, by Professor Hazen’s group, have also demonstrated that urinary bromotyrosine has value in the prediction of exacerbation as well as corticosteroid resistance in children with asthma [11], [12]. Future studies are required to compare the predictive value of urinary bromo-HPA *versus* bromotyrosine in the assessment and monitoring of patients with asthma. We also found that HPA is the major urinary metabolite of bromotyrosine. However, this de-brominated compound is not exclusively associated with bromotyrosine metabolism and HPA is a common end-product of tyrosine, and chlorotyrosine metabolism [9]. Although increased urinary excretion of HPA has been reported in a variety of medical conditions [21], increased urinary HPA cannot be used to specifically assess either bromination or chlorination reactions *in vivo*.

Amino acids have a large volume of distribution (V_d_) in the body and they can enter the cells and be used as the building blocks for protein synthesis. Less than 2% of injected [D_3_]bromotyrosine as its urinary metabolites was recovered over the 24 h following its injection. This finding corroborates with the large volume of distribution of bromotyrosine *in vivo*. However, we did not know, whether or not, free bromotyrosine can be used for cellular protein synthesis. Therefore, HepG2 cells were incubated with deuterium labeled bromotyrosine and protein-bound [D_3_]bromotyrosine and [D_3_]tyrosine in the cellular fractions were measured 24 h after incubation with [D_3_]bromotyrosine. Although no protein-bound [D_3_]bromotyrosine was found in the cell lysate, we were able to detect [D_3_]tyrosine in the cell fraction which corroborates with the de-bromination of bromotyrosine and incorporation of deuterium-labeled tyrosine in the cellular proteins. We have previously reported similar findings after incubation of cultured cells with labeled chlorotyrosine [9]. This indicates that cellular de-halogenation is not restricted to de-chlorination and de-iodination reactions [9], [19], [20]. We have previously ruled out the role of cytochrome P450 de-chlorination of chlorotyrosine [9]. Although the metabolism of chlorotyrosine and bromotyrosine seems to be similar, the formation and metabolism of these products could follow different kinetics [22], [23], [24]. In a previous report we were unable to show a significant de-chlorination of chlorotyrosine by DEHAL1 *in vitro* [9]. In present study we tested whether or not bromotyrosine, di-bromotyrosine, or bromo-HPA can interact with DEHAL1 and found out that DEHAL1 can catalyse de-bromination of bromotyrosine and di-bromotyrosine *in vitro.* This data corroborates with previous report by McTamney and Rokita who showed that bromotyrosine is capable of oxidizing reduced DEHAL1 under the same condition described for iodotyrosine [23]. Our results also indicated that brominated tyrosine (but not bromo-HPA) can inhibit deiodinase activity of DEHAL1 *in vitro*. Our report indicates that DEHAL1 is also a de-brominase enzyme and its activity is not restricted to de-iodination of iodotyrosine, as typically described (*i.e*. iodotyrosine deiodinase). DEHAL1 is expressed in a variety of tissues (including the thyroid gland, liver and kidney) and plays an important role in recycling of iodide in mammalian tissues [19], [20]. Although a dramatic reduction in DEHAL1 activity is known to cause congenital hypothyroidism [19], *in silico* studies have shown that partial inhibition of DEHAL1 activity may lead to abnormal thyroid function [25]. Inhibition of iodotyrosine deiodinase activity by bromotyrosine (a by-product of leukocyte activation) may contribute to thyroid abnormalities that are associated with systemic inflammation [25]. However, further investigations are required to examine the role of bromotyrosine in pathogenesis of thyroid disorders.

Bromine is mostly present in animals as ionic bromide and, apart from its role in eosinophils, it serves an essential function in the formation of sulfilimine crosslinks in collagen type IV [26]. Since this process requires the formation of hypobromous acid, bromination of the extracellular matrix might be considered as a by-product in the development of essential crosslinks of the extracellular matrix. Likewise, Baldus *et al.* reported that transcytosis of myeloperoxidase confers the specificity to modification of the tyrosine-residues of vascular matrix proteins during systemic inflammation [11]. Therefore, it is likely that a de-bromination or de-chlorination mechanism may play a protective role during tissue regeneration following inflammation. We have previously demonstrated that rat liver homogenates can de-chlorinate protein-bound chlorotyrosine *in vitro* [9]. Kamisaki et al. also reported a “denitrase” activity that catalyzed de-nitration of protein-bound nitrotyrosine *in vitro* [27]. We did not study the de-bromination of protein-bound bromotyrosine in the present study. This can be further investigated, along with the mechanism of de-chlorination and de-nitration in future.

One of the limitations of our study is that we did not investigate the mechanism of bromo-HPA formation in our experimental model. It is not known, whether or not, bromo-HPA is exclusively derived from the metabolism of bromotyrosine. Fu et al., demonstrated that reaction of free chlorotyrosine with HOCl may lead to the formation of chloro-hydroxyphenylacetaldehyde, which is structurally similar to (but distinct from) chloro-HPA [28]. Such reaction may occur for bromotyrosine as well, and needs to be explored in future investigations. Likewise, we previously reported that nitro-HPA is not exclusively derived from nitrotyrosine metabolism, and it is mainly formed by nitration of HPA in both humans and rats [14], [29]. To determine whether or not bromo-HPA could be formed by endogenous bromination of HPA in healthy rats, deuterium-labeled HPA ([D_6_]HPA) was synthesized and injected into healthy rats as described [14]. The formation of deuterated bromo-HPA was then monitored in urine samples. We were unable to detect [D_5_]bromo-HPA in rat urine following intraperitoneal injection of [D_6_]HPA in healthy rats (data not shown). This may indicate that bromination of endogenous HPA does not occur abundantly *in vivo*. Another limitation of the present study is that we did not test whether or not bromo-HPA can undergo de-bromination *in vivo*. However, our *in vitro* study showed that unlike bromotyrosine, incubation of bromo-HPA with either DEHAL1 or rat liver homogenates was not associated with formation of de-brominated products (data not shown).

In conclusion, we identified di-bromo-HPA and bromo-HPA as the main brominated urinary metabolites of di-bromotyrosine and bromotyrosine in rats. However, de-halogenation of bromotyrosine also occurs during metabolism of bromotyrosine *in vivo*.

## Conflict of interest

None.

## Figures and Tables

**Table 1:** The mass balance of the derivatized authentic, ^13^C and deuterium-labeled compounds that were used for single-ion monitoring during mass spectrometry.

**Compound**	**Authentic**	^**13**^**C-labeled**	**Deuterium-labeled**
Tyrosine	Tyrosine	[^13^C_9_]Tyrosine	[D_3_]Tyrosine
407	415	410
3-Bromotyrosine	Bromotyrosine	[^13^C_9_]Bromotyrosine	[D_3_]Bromotyrosine
489	498	492
4-Hydroxyphenylacetic acid (HPA)	HPA	[^13^C_8_]HPA	[D_3_]HPA
331	339	334
3-Bromo-HPA	Bromo-HPA	[^13^C_8_]Bromo-HPA	[D_3_]Bromo-HPA
409	417	412
3,5-Di-bromo-HPA	Di-bromo-HPA	[^13^C_8_]Di-bromo-HPA	Not used in this study
489	497	

**Table 2:** Urinary excretion of deuterated bromotyrosine, tyrosine, bromo-HPA and HPA after intraperitoneal injection of 500 nmole of [D_3_]bromotyrosine in rats.

	**[D**_**3**_**]Bromotyrosine**	**[D**_**3**_**]Tyrosine**	**[D**_**3**_**]Bromo-HPA**	**[D**_**3**_**]HPA**
Total amounts (nmole) excreted in 24-h urine	0.62±0.10	Undetectable	2.15±0.21	6.66±1.33
Molar percentage of the excreted metabolites in 24-h urine	0.12±0.02%	Undetectable	0.43±0.04%	1.33±0.26%
